# p53 signaling in cancer progression and therapy

**DOI:** 10.1186/s12935-021-02396-8

**Published:** 2021-12-24

**Authors:** Hany E. Marei, Asmaa Althani, Nahla Afifi, Anwarul Hasan, Thomas Caceci, Giacomo Pozzoli, Andrea Morrione, Antonio Giordano, Carlo Cenciarelli

**Affiliations:** 1 Department of Cytology and Histology, Faculty of Veterinary Medicine, Mansoura University, Mansoura, 35116 Egypt; 2 Biomedical Research Center, Qatar University, Doha, Qatar; 3 Qatar Biobank, Doha, Qatar; 4 Department of Mechanical and Industrial Engineering, College of Engineering, Qatar University, Doha, Qatar; 5 Biomedical Sciences, Virginia Maryland College of Veterinary Medicine, Blacksburg, VA USA; 6 Pharmacology Unit, Fondazione Policlinico A. Gemelli, IRCCS, Rome, Italy; 7 Sbarro Institute for Cancer Research and Molecular Medicine. Center for Biotechnology, College of Science and Technology, Temple University, Philadelphia, PA USA; 8 Department of Medical Biotechnology, University of Siena, Siena, Italy; 9 Institute of Translational Pharmacology-CNR, Rome, Italy

**Keywords:** p53 signaling, Tumor suppressor gene, Gain of function mutation, Cancer progression, Cancer therapy

## Abstract

The p53 protein is a transcription factor known as the "guardian of the genome" because of its critical function in preserving genomic integrity. The *TP53* gene is mutated in approximately half of all human malignancies, including those of the breast, colon, lung, liver, prostate, bladder, and skin. When DNA damage occurs, the *TP53* gene on human chromosome 17 stops the cell cycle. If p53 protein is mutated, the cell cycle is unrestricted and the damaged DNA is replicated, resulting in uncontrolled cell proliferation and cancer tumours. Tumor-associated p53 mutations are usually associated with phenotypes distinct from those caused by the loss of the tumor-suppressing function exerted by wild-type p53protein. Many of these mutant p53 proteins have oncogenic characteristics, and therefore modulate the ability of cancer cells to proliferate, escape apoptosis, invade and metastasize. Because p53 deficiency is so common in human cancer, this protein is an excellent option for cancer treatment. In this review, we will discuss some of the molecular pathways by which mutant p53 proteins might perform their oncogenic activities, as well as prospective treatment methods based on restoring tumor suppressive p53 functions.

## Introduction

Tumor suppression is the main function of p53 protein, which is encoded by the *TP53* gene on human chromosome 17. The p53 protein is posited to inhibit the phenotypic and genomic alterations associated with cancer development through a complex interplay with several signaling pathways known to play critical roles in essential cellular processes such as cell division, maintenance of genomic stability, apoptosis, autophagy, immune response, and regulation of tumor microenvironment (TME) [1, 2].

Binding of wild-type p53 protein to specific DNA response elements induces the expression of a wide array of genes that ultimately guard against cancer development and progression [3]. Under physiological conditions, exposure of cells to different stress signals activates the p53 signaling pathway, allowing the cells to activate several transcriptional programs including cell cycle arrest, DNA repair, senescence, and apoptosis leading to suppression of tumor growth [4].

In most if not all human malignancies, inactivation of the *TP53* gene usually occurs through the acquisition of loss of function mutations or negative regulation of wild-type p53 proteins. Inactivation of the *TP53* gene drives invasion, proliferation, and cell survival, thereby facilitating cancer progression and metastasis [5]. More than 75% of *TP53* gene mutations result in loss of wild-type p53's activities. Mutated p53 proteins might act either as dominant negative over wild-type p53 action [6], or gain novel tumorigenic properties counteracting the protective function of wild-type p53 [7, 8].

Two homologs of the tumour suppressive transcription factor p53, p73 and p63, play crucial roles in cancer development. Because the p53 family members have a lot of structural similarities, p73 and p63 can bind to most p53-responsive promoters and initiate transcription. Apart from shared functions with p53 (e.g., activation of apoptosis in response to cellular stress), structural variability within the family has given p63 and p73 different responsibilities. The p53 family members p73 and p63 play overlapping and distinct roles in development and homeostasis. The functional linkages between family members can be better appreciated by looking at the expression of the p73 and p63 isoforms in human tissue. *TP63* expression has been found to be significantly related across tissues. In tissues with concurrent mRNA expression, nuclear co-expression of both proteins was detected in the majority of cells [9].

Here we will highlight recent advances in the understanding the regulatory network by which mutant p53 proteins might modulate molecular signaling pathways involved in cancer progression and/or protection.

### p53 and cancer progression

**Fig. 1:**
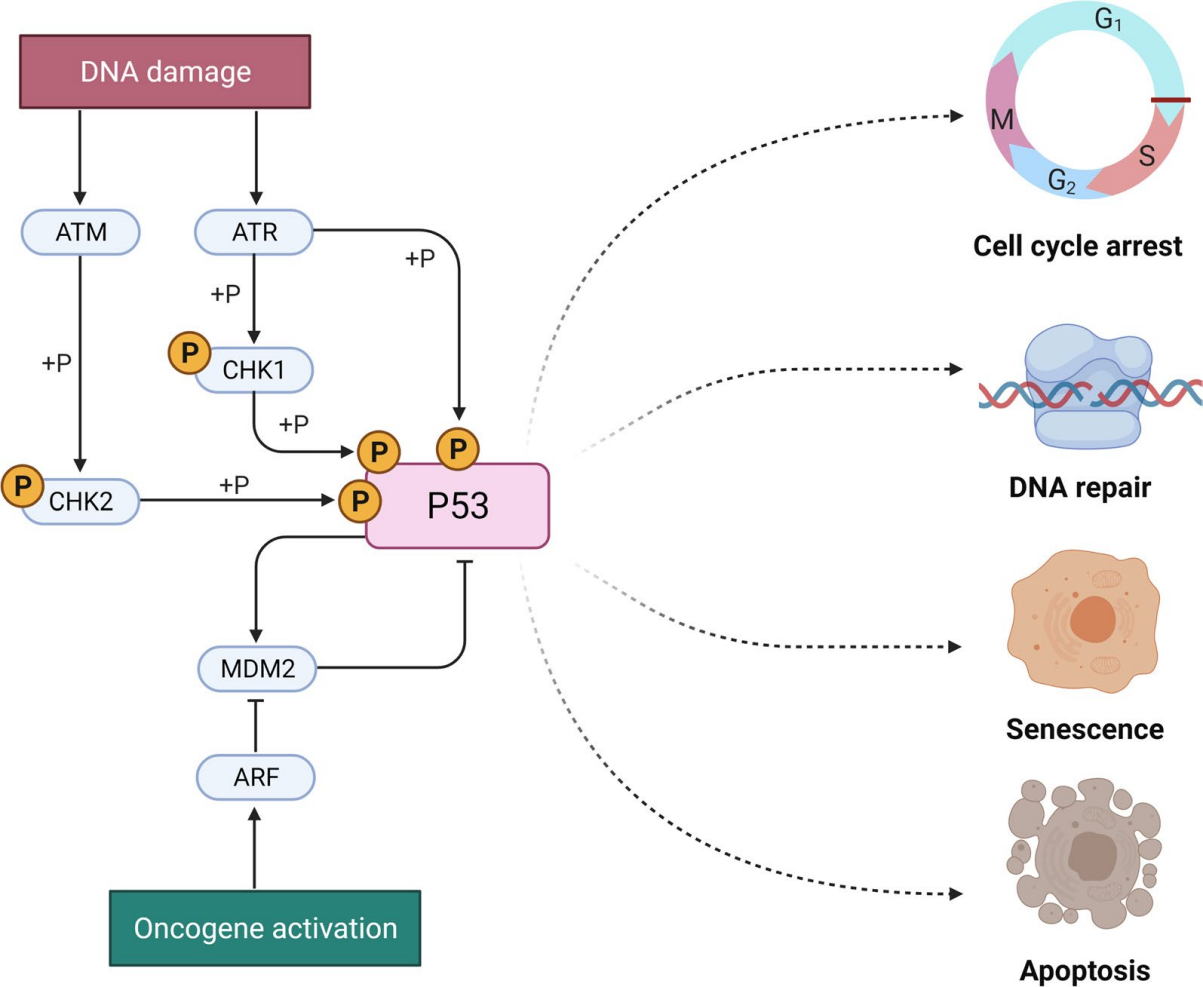
*ATM* (ataxia telangiectasia mutated) and ataxia-telangiectasia-mutated-and-Rad3-related kinase (ATR) protein kinases phosphorylate p53 at serine 15 to activate and enhance the p53 stability. The phosphorylation of a variety of substrates, including casein kinase (CK1), checkpoint kinase 1 (Chk2), and p53, regulates cancer cell viability by modulating many critical biochemical pathways that lead to cell cycle arrest, DNA repair, senescence, and death

Mutation in the *TP53* gene is detectable in about 50% of human breast, colon, lung, liver, prostate, bladder, and skin cancer. Upon DNA damage, wildtype p53 acts in restraining the process of cell replication until the damage is repaired, thus preventing the propagation of DNA-defective cells and the acquisition of a cancer phenotype (Fig. 1). On the other end, *TP53* mutations affect cell cycle and cells loose control of cell proliferation leading to propagation of damaged DNA into their progenies, which become transformed into cancerous cells.

The wild-type *TP53* gene is translated into p53 proteins, which are transcription factors with an important role in orchestrating a variety of cellular responses such as DNA repair, cell cycle arrest, cellular senescence, cell death, cell differentiation, and metabolism thereby driving inhibitory molecular processes on cancer growth (Fig. 1) preserving genomic integrity, thus acting as "guardian of the genome" [10, 11].

Nuclear p53 phosphoprotein regulates normal cell proliferation, and accumulation of non-functional mutated p53 in tumor cell nuclei is associated with the development and/or progression of several neoplastic diseases, including breast cancer [12]. Activation of p53 induces senescence and cell-cycle arrest under excessive oncogenic stress and this is a crucial mechanism regulating the inhibition of tumorigenesis. Oncogenic cues often converge on key signaling nodes involved in the regulation of mTOR kinase [13]. Cell proliferation is induced by inactivating the p53/p21 Cdk-interacting protein 1 (Cip1) complex via Ras-dependent and-independent stimulation of the Raf/MEK/ERK cascade. These findings point to the importance Ras oncogenic activity and p53 inactivation in human cancer. [14]. The p53/p21/p27 and p53/Bcl-2/Bax pathways affect many biological processes including cell proliferation, G2/M phase and apoptosis [15] as in fact 53 tumor suppressive function might work through the recruitment or regulation of other tumor suppressor proteins such as the Inter-Alpha-Trypsin Inhibitor Heavy Chain 5 proyein, encoded by the *ITIH5* gene. p53 directly binds to the promoter of *ITIH5* in melanoma cells, promoting ITIH5 transcription and therefore suppressing melanoma cell proliferation and migration, likely by downregulating KLF4 transcriptional activity [16]. ZEB1 and ZEB2 are transcription factors that induce epithelial–mesenchymal transition (EMT), while p53 inhibits EMT by suppressing ZEB1 and ZEB2 expression (Fig. 3) [17].

### Mechanism of action of mutant p53

#### Mutant p53 and modulation of wild-type p53 function

In contrast to wild-type p53 anti-tumor protective activity, mutant p53 proteins have oncogenic action in culture cells [18], and promote metastasis and genomic instability in mice models [19, 20]. Mutations of p53 are often alterations in the central DNA-binding domain and several hotspots such as R175, G245, R248, R249, R273 and R282, have been so far identified [21]. p53 mutations are subdivided into two main categories—structural and DNA-contact mutations, which affect either folding of the p53 protein or the transcriptional activity of p53 and regulation of target genes, respectively. In both cases, the structural stability of p53 is altered and p53 might acquire a gain/loss-of-function phenotype [22, 23]. One of the major consequences of p53 mutant activities is altered gene expression, and in most cases, their ability to bind canonical p53 elements is severely hampered [24]. The interaction of mutant p53 proteins with non-canonical/different response elements might induce an oncogenic response as in fact mutant p53 proteins might act as a tumor-initiating transcriptional factors [25]. Mutant p53 proteins may also control gene expression through different mechanisms in which they interact directly with DNA sequences that bind to nuclear matrix regions, giving another way to change/regulate gene expression [26].

#### Binding/interaction of mutant p53 with key transcription factors including p63/p73 axis

The interaction with the nuclear factor Y (NF-Y) which occurs in response to minor DNA damage, and dysregulates cycle checkpoints [27–31]. Mutant p53 proteins can also interact with other transcription factors to induce inhibitory responses [32]. The mechanisms by which p53 mutants control the functions and downstream effector genes of p63 and p73 family members, is intriguing and represent one of the best understood process relevant to the p53-transcription factor function [33, 34]. The existence of several isoforms further complicates this process, and interestingly, mutant p53 interaction with various isoforms of p63 and p73 was shown to increase the expression of p63 downstream genes [31, 35]. Other transcriptional factors such as TopBP1and PIN1 promote or enhance binding of mutant p53 to p63, respectively [36, 37].

Published data reported that mutant p53 increases the ability to develop spontaneous metastasis in mice by inhibiting p63 and p73 functions [38]. Loss of p63 and/or p73 activities is linked to the development of spontaneous tumors and the capacity of cancer cells to invade [36, 39, 40]. Modulation of the invasive ability of cancer cells is controlled by multiple p63 target genes, which are affected by the interaction between mutant p53 and p63. The mutant p53/Smad complex, for example, inhibits p63 and promote TGF-induced metastasis [36]. A Pin1/mutant p53 axis evokes aggressiveness in breast cancer through the inhibition of p63-regulated expression of Dicer [34, 39], and p53 mutants can prevent DEPDC1 (DEP domain containing 1) gene inhibition mediated by p63 [34]. The interaction of mutant p53 and p63 modulates cell migration and invasion by promoting recycling and signaling of cell surface receptors such as the epithelial growth factor receptor (EGFR) and the hepatocyte growth factor receptor (HGFR) [41]. Recruitment of diacylglycerol kinase (DGKα) in the invadopodia of migrating cells has been reported to enhance the p53/p63/RCP-dependent invasion mechanisms [42]. Furthermore, p73 has a similar role to p63, and it plays a decisive role in enhancing key cellular processes including cell aging and apoptosis [43].

#### Mutant p53 and other regulatory mechanisms

Mutant p53 also targets other regulatory molecules including microRNAs such as miR-130b, miR-155 and miR-205. p53 binding to microRNAs has been associated with not only altering the stability of those molecules, but also influencing crucial molecular pathways involved in invasion and metastasis through the modulation of transcripts such as *ZEB1* and *ZNF652 *[44, 45]*.* One notable consequence is the existence of mutant p53 in many cancer types is usually associated with gain of invasive and metastatic activity. For example in non-small lung cancer (NSLC), low p53 expression and high PGC1 expression were linked to a shorter survival time in NSCLC patients [46].

Mutant p53 additionally regulates certain signaling cascades such as the mevalonate pathway which regulate tissue remodeling [47]. However, how mutant p53 interacts with diverse partner molecules and how this connection is linked to distinct functional role as gain-of-function or loss-of-function is still not totally understood. Amongthe different domains constituting the molecular structure of mutant p53, the N- and C- termini regulate the interaction of mutant p53 with p63 and p73 as well as other molecules. Specifically, the DNA-binding domain and C-terminus of mutant p53 inhibit p63 function, and play crucial roles in regulating cell invasion and apoptosis [37, 48]. Moreover, the interaction of the N-terminus domain of the mutant p53 is crucial for inducing the expression of target genes such *GRO1 and CXCL1* and transcriptional factors such as sterol regulatory element binding proteins (SREBPs) and thus it has been reported to modulate the drug-induced apoptosis [26, 27].

#### Binding/interaction of mutant p53 with other protein molecules

In addition to the interactions with multiple transcription factors, mutant p53 proteins bind and regulate the function of other non-transcription factor proteins. In this regard, mutant p53 disrupts DNA-repair mechanisms by interacting with the DNA nuclease MRE11 [49]. Moreover, the interaction of mutant p53 with other proteins involved in cell cycle regulation such as BTG2 modulates H-Ras, thereby enhancing oncogenic transformation [50]. Other mutant p53 targets are involved in the regulation of genomic stability including the topoisomerase 1 (Top1), and the binding of mutant p53 to Top1 led to loss of negative regulation induced by the wild-type p53 and induced hyper-recombination and genomic instability [51].

#### Posttranscriptional modifications of mutant p53

p53 protein has a rapid turnover due to ubiquitination mediated by the E3 ubiquitin ligase MDM2 and subsequent proteasomal degradation [52, 53].

p53 stability in response to genotoxic stress is regulated by a variety of post-translational modification (PTMs) [54], including phosphorylation, acetylation, methylation, glycosylation, ubiquitination, and sumoylation, which occur in different regions of the p53 protein, and play crucial role in regulating of p53 stability and localization, thereby affecting p53 ability to modulate cell proliferation and cell death [55].

##### Phosphorylation of p53 and its role in cancer progression and apoptosis regulation

Different serine (S) and threonine (T) phosphorylation sites have been identified on p53 proteins particularly in the C- and N-terminal domains [56]. Notably, Ser 15 phosphorylation is pivotal in the activation of p53 [57] as in fact it enhances p53 stability by promoting its dissociation from the MDM2 ubiquitin ligase [58]. *ATM* (ataxia telangiectasia mutated) and ataxia-telangiectasia-mutated-and-Rad3-related kinase (ATR) protein kinases [54, 55] phosphorylate p53 at serine 15 and activate and improve the stability of p53 by enhancing the interaction between p53 and histone/lysine acetyltransferase (HATS) [59]. ATM- and ATR-mediated phosphorylation of downstream substrates, including casein kinase (CK1), checkpoint kinase 1 (Chk2), and p53, limits cancer cell viability by modulating many critical biochemical pathways leading to cell cycle arrest, DNA repair, senescence, and death. [60].

**Fig. 2:**
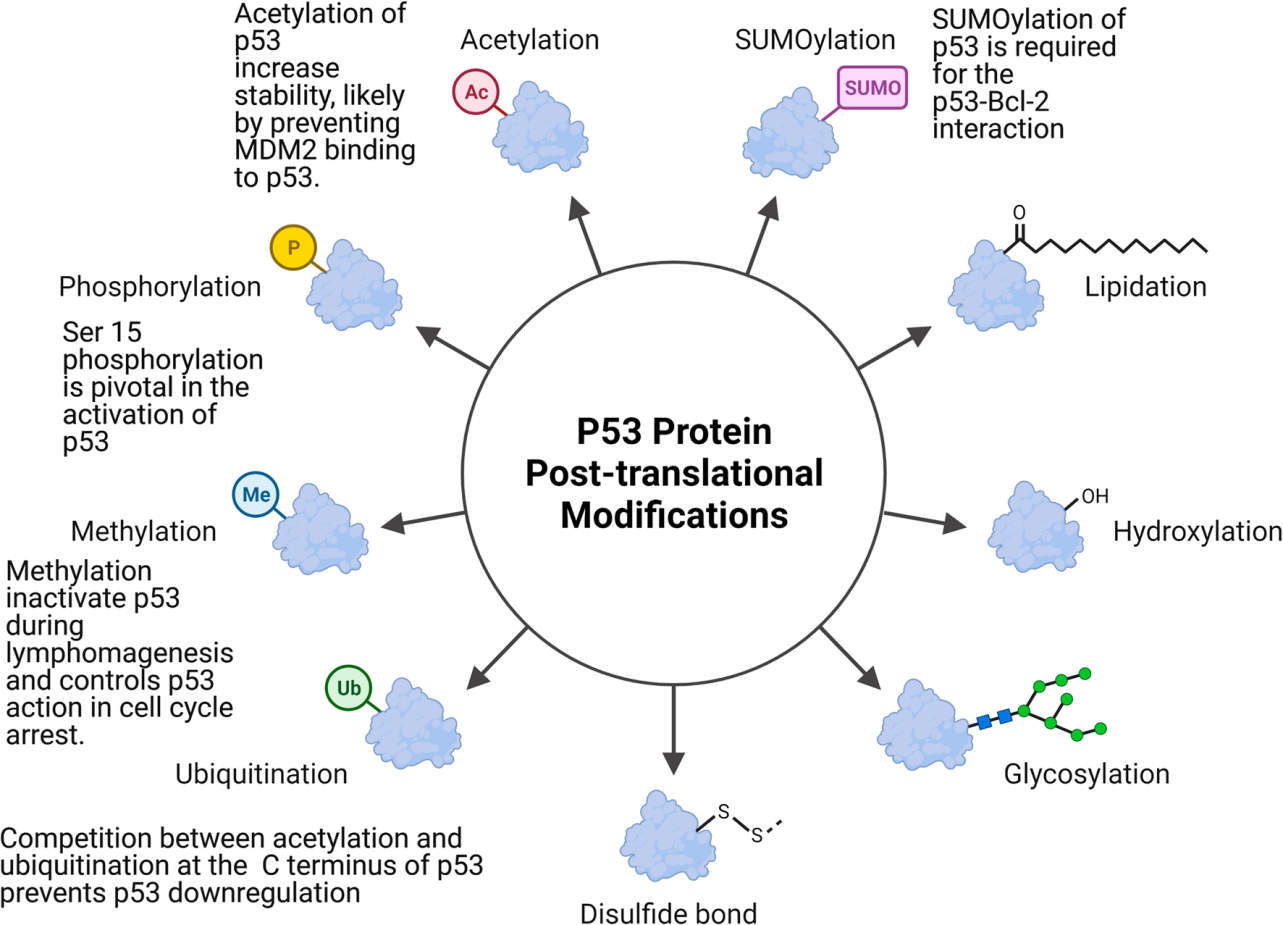
Phosphorylation, acetylation, methylation, glycosylation, ubiquitination, and sumoylation occur in different regions of the p53 protein and play a crucial role in regulating p53 stability and localization in response to genotoxic stress. p53 ubiquitination mostly occurs at its C terminus, where acetylation also occurs during cell stress and by competing with ubiquitination it prevents p53 downregulation, thus enhancing p53 stability

Phosphorylation of p53 on other serine residues activates/modulates p53 functions, and it associated with cell exposure to different toxic or damaging stimuli such as UV and IR (Fig. 2). DNA damage after UV and IR exposure mediates stabilization of human p53 through phosphorylation on Ser-20 [61]. Apoptosis is triggered instead by p53 phosphorylation on Ser46, which is mediated by many kinases including homeodomain-interacting protein kinase 2 (HIPK2), p38, and dual specificity tyrosine-phosphorylation regulated kinase 2 (DYRK2) [62, 63]. Genotoxic stress causes transactivation of p53 by modulating p53 phosphorylation on its amino terminus, which regulates the interactions between p53 and MDM2 orp300/CBP [64, 65]. The interactions of p53 with the apoptosis stimulating proteins of p53 (ASPP) proteins, as well as p300, control its apoptotic activity [66, 67].

##### Acetylation of p53 and its effects on p53 transactivation and stability

Acetylation of p53 is another post-translational reversible enzymatic process by which p53 action is fine-tuned in response to different cellular toxic signals including genotoxic stress and damage of DNA [68] and many studies have looked at the role that acetylation plays in regulating p53 action as a tumor suppressor (Fig. 2) [68].

p53 proteins is are acetylated on at defined amino acid positions. Particularly, lysines (K) residues within the C-terminal regulatory domain (K370, K372, K373, K381, K382, and K386) of p53 are key acetylation sites and play a crucial role in regulating p53 function [69, 70]. Acetylation of the aforementioned lysine residues modulate p53 transcriptional activity and increase stability, likely by preventing MDM2 binding to 53 [56].

The transcriptional coactivator proteins CBP/p300 control p53 activity in multiple ways., interaction between p300 and either p53 or E2F1 has a significant influence on early cell cycle progression, suggesting that p300 is involved in E2F and p53-regulated growth arrest pathways [71]. CBP/p300 proteins also contributes to MDM2-mediated ubiquitination of p53, which reduces p53 levels in the presence of genotoxic stress [72]. On the other hand, they also prevent p53 degradation by acetylating the protein's carboxyl terminus, which includes ubiquitination sites. Notably, K120 and K164 acetylation sites are located in the p53 DNA-binding domain, which is the most commonly mutated region of p53 in solid tumors, A K120 mutation was identified in Ewing's Sarcoma and esophageal SCC cells, whereas a K164 mutation was discovered in glioblastoma and bladder cancer cells [70] suggesting that acetylation of these particular residues is critical for p53 function.

##### The tumor suppressor action of p53 is modulated by methylation

P53 lysine (K) and arginine (R) residues can be methylated, and a growing number of studies have shown that p53 methylation occurs during the DNA damage response [73]. The effects of lysine and arginine methylation on chromatin structure and gene expression have been well characterized [74–76] In the tetramerization domain, PRMT5 methylates p53 at different arginine residues (R333, R335, and R337) [77], and this modifications likely inactivate p53 during lymphomagenesis and controls p53 action in cell cycle arrest (Fig. 2) [78]. SET and MYND domain-containing protein 2 (SMYD2) monomethylate p53 at K370 and decrease p53-mediated transactivation. In addition, this methylation event reduces p53 binding to the promoters of its target genes, such as p21 [79]. SET7/9-evoked monomethylation of K372 enhances p53-mediated activation of downstream targets, whereas SET8-mediated monomethylation of K370 reduces p53 transcriptional activity [80].

##### SUMOylation of p53 controls its localization

The tumor suppressor p53 has dynamic nuclear output because its tetramer domain contains a leucine-rich nuclear export signal (NES) region [81]. Another NES is recognizable in the N-terminal transactivation domain of p53 and its phosphorylation blocks p53 nuclear output, leading to p53 nuclear accumulation [82]. SUMO-1, SUMO-2 and SUMO-3 promote sumoylation of p53 at K386, which accelerates p53 nucleocytoplasmic shuttling (Fig. 2) [83].

P53 promotes the synthesis of pro-apoptotic genes in the nucleus by increasing p21 expression, [84]. The majority of the anti-apoptotic activities of p53 occurs in the nucleus, particularly while the cell is at rest.

Topors and other members of the PIAS family SUMOylate the p53 protein at a single location, K386, on the protein. [85]. When PAISy was administered to p53, K386 sumoylation and K120 acetylation of p53 occurred, and Tip60 was activated as a result. Despite the fact that these two changes are not mutually exclusive, they act as "binary death signals," causing p53 cytoplasm accumulation and PUMA-mediated autophagy. [86].

The growth suppressive action of p53 is lost when it is shuttled into the nucleus, where it may instead promote cellular proliferation. Cytosolic p53 has a non-transcriptional function and. Can interact with Bcl (B cell lymphoma/leukemia)-2, thereby counteracting its anti-apoptotic impact [87]. Furthermore, SUMOylation of p53 is required for the p53-Bcl-2 interaction [88] and high levels of cytoplasmic p53 localization is associated with poor prognosis and hormone-resistant disease [89].

##### Ubiquitination and ubiquitin-like proteins that impact on the p53 pathway

Ubiquitination plays an important role in regulating protein function as in fact it modulates proteins trafficking, localization, stability and activity. It also has an important role in regulating protein-to-protein interactions [90]. The role of ubiquitination in regulating transcriptional factor function has lately received a lot of attention. p53 ubiquitination mostly occurs at its C terminus, where acetylation also occurs during cell stress and by competing with ubiquitination it prevents p53 downregulation, thus enhancing p53 stability (Fig. 2) [91]. However, the impact of ubiquitination on the function of the P53 tumor suppressor is very broad and beyond the scope of this article. Recent review papers, such as those by Chen et al., nicely cover this topic [92].

### Mutant p53 and other cancer related signaling pathways

#### 0.1 Mutant p53 and STAT signaling pathway

Through a mechanism involving Stat3, which binds to the p53 promoter in vitro and in vivo, oncogenic signaling pathways decrease the rate of p53 gene transcription. STAT3-induced inhibition is partially abrogated by a site-specific mutation of a STAT3 DNA-binding site in the p53 promoter. STAT3-induced inhibition is partially abrogated by a site-specific mutation of a STAT3 DNA-binding site in the p53 promoter. STAT3 activity also has an impact on the p53 response genes and UV-induced cell growth arrest in normal cells. Furthermore, inhibiting STAT3 in cancer cells increases p53 expression, resulting in tumour cell death mediated by p53 [93–95]. STAT3, like STAT5 and STAT6, affects the tumor microenvironment (TME), boosting immunosuppressive TMEs and decreasing anti-tumor immunity [96].

STAT proteins have an important role in regulating p53 activation. STAT3 reduces the tumor-suppressive action of p53 by inhibiting its expression [97]. As shown in breast [98] and prostate cancer cells [99], wild-type p53 affects tyrosine phosphorylation and hence limits STAT3 DNA-binding activity in a manner that resembles a feed-back loop.

**Fig. 3:**
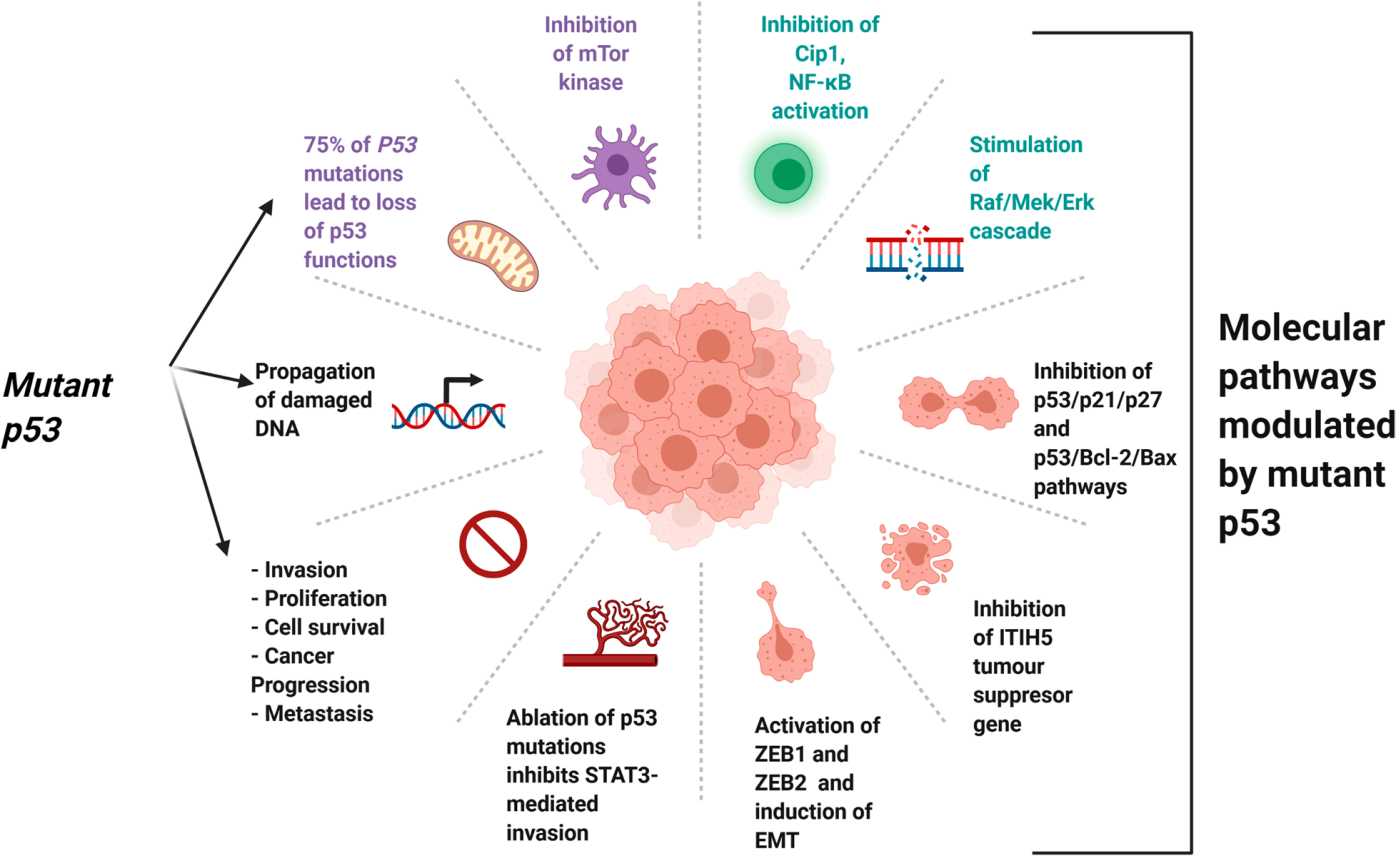
Different p53 mutations and their potential effects on its function and oncogenic activity. Mutation of *TP53* genes is associated with inactivation of wild-type *TP53* gene and 75% of *P53* mutations lead to loss of p53 functions. Inactivation of the wild type function of p53 promotes invasion, proliferation, cell survival, cancer progression, and metastasis. The effects of p53 mutations are mediated by interaction with key molecular pathways that includes: Inhibition of mTor kinase, inhibition of Cip1, NF-κB activation, stimulation of Raf/MEK/ERK cascade, inhibition of p53/p21/p27 and p53/Bcl-2/Bax pathways, inhibition of ITIH5 tumour suppressor gene, activation of ZEB1 and ZEB2 transcription factors and induction of epithelial–mesenchymal transition. Ablation of R248Q p53 mutation in CRC inhibits STAT3-mediated tumor growth and invasion. The net effects of p53 mutations is the loss of the protective effects of wild p53 proteins which lead to cancer progression

This reciprocal regulation between activated STAT3 forms and p53 does not occur when p53 is mutated (Fig. 3). The ability of phosphorylated or alternatively spliced STAT3 proteins to increase mutant p53 expression might pose a risk for cancer. Notably, constitutive STAT3 activation is only detectable in cancer cells expressing inactivating mutations or deletions of p53, allowing cancer cells to evade inhibition by the wild-type p53 pathway, particularly after DNA damage. The presence of STAT3 and p53 in cancer cell lines from prostate (DU145 and TSU), breast (MDA-MB-468 and SK-BR-3) and ovarian (MDAH 2774, SKOV-3, and CAOV-3) cancer confirms this theory (which [99]. These cell lines express constitutively active STAT3 proteins in conjunction with mutant p53 or p53 null background. According to a recent study, the R248Q p53 mutation is associated with hyperactive STAT3/JAK signaling, and therapeutic ablation of this gain-of-function mutant p53 in colorectal cancer inhibits STAT3-mediated tumor growth and invasion [100].

In colorectal cancer (CRC), the frequent hotspot mutations mutp53R248Q and mutp53R248W exhibit gain-of-function activities and constitutively bind to and activate STAT3 thereby enhancing proliferation and invasion in CRC mouse model. In mutp53R248W-expressing PDAC cells, genetic or pharmacological STAT3 depletion phenocopied mutp53 depletion and decreased cell viability and migration indicating that the mutp53R248W signals through the STAT3 transforming axis [101]. Modulation of STAT3-dependent gene expression altered biological responses, including cell cycle progression and p53 response [102].

##### Mutant p53 and NF-kappaB (NF-κB) signaling pathway

The NF-κB and p53 pathways are often seen as antagonistic transcriptional networks. While wild-type p53 traditional function is growth restriction, NF-κB's promotes cell survival and inflammation [103]. However, these two pathways can cross-talk and cooperate in determining similar biological responses [104]. NF-κB cooperates with wild-type p53 in mediating apoptosis of IMR-90 cells but not of human BJ fibroblasts [105] and functionally interact with wild-type p53 in promoting senescence [106]. In addition, in macrophages and monocytes NF-κB is necessary for p53-dependent regulation of many pro-inflammatory genes in order to enhance tissue and inflammatory responses to damaging signals [107].

In mutant p53 animal models, Cooks et al. discovered that mutant p53, in combination with tumor necrosis factor (TNF), prolongs NF-κB activation, resulting in a chronic inflammatory phenotype and colon cancer growth. These data support a connection between accumulating missense p53 mutations and NF-κB activation in human cancers linked with colitis [108].

The combined binding of NF-κB, the R273H p53 mutant, and other mutant versions of p53 to these enhancers regulates RNA polymerase II recruitment to these elements in colorectal carcinomas, boosting mRNA synthesis and activation of tumor-promoting genes. Mutant p53, in combination with NF-κB, can therefore alter the inflammatory tumor microenvironment (TME), inducing in both epithelial and and non-epithelial cells the expression of cancer-promoting gene (Fig. 3) [107]. Using 9-aminoacridine derivatives in renal cell carcinomas and small molecule curaxins in several cancer cell lines and mouse tumour xenografts to restore wild-typep53 function is a rational approach that has previously been demonstrated [109, 110]. In mutant p53 background, approaches aiming at restoring wild-type p53 expression might potentially improve existing NF-κB-dependent therapies [111].

#### Mutant p53 and tumor microenvironment (TME)

**Fig. 4:**
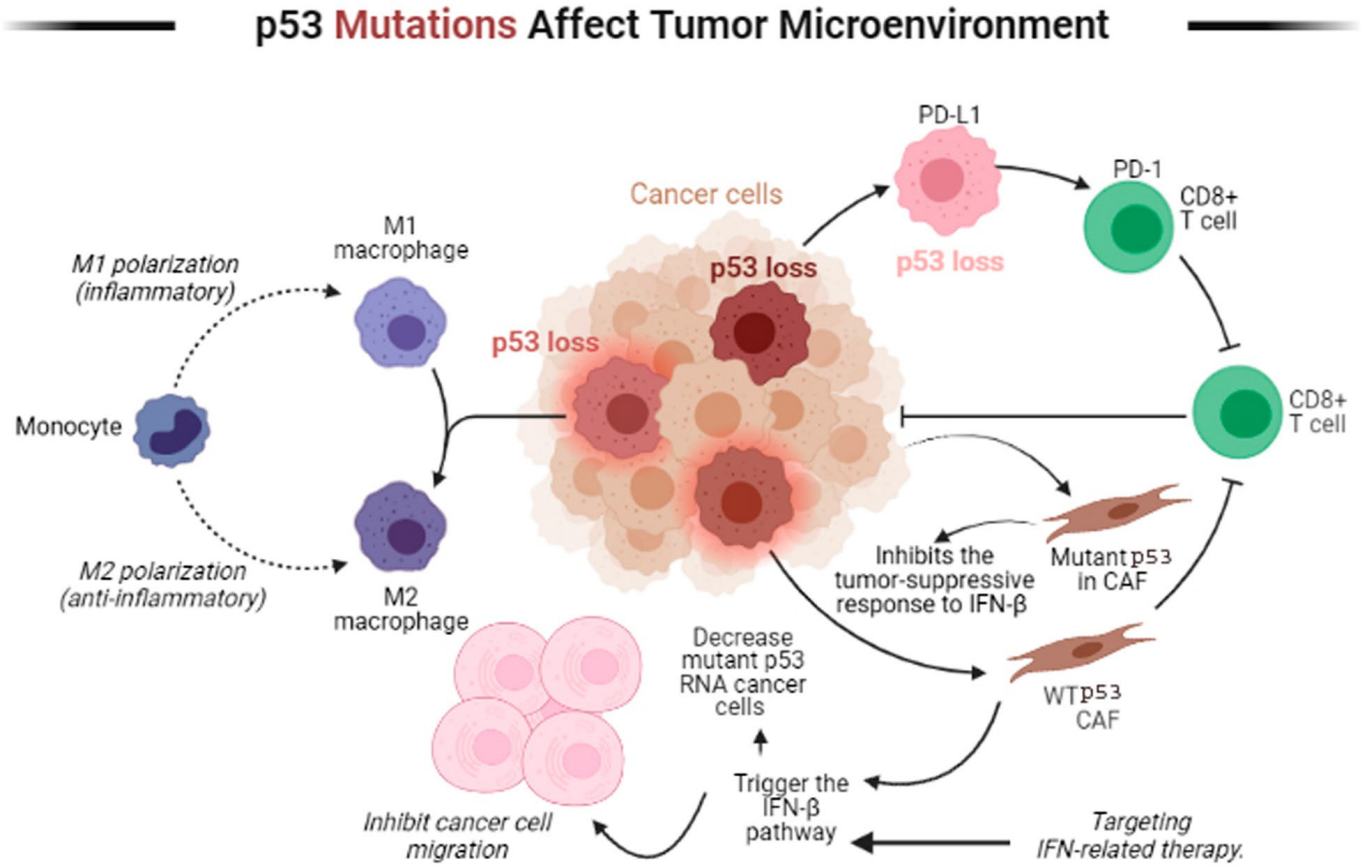
Mutant p53-expressing tumors can reprogram M2-type macrophages (M2) and increase tumor invasion. High wild-type p53 activity acts as a brake on M1-like macrophage and, decreased M1-like gene expression. When cancer associated fibroblasts (CAFs) come into contact with cancer cells, their Interferon-β pathway is triggered and interacts with wild-type p53 in fibroblasts to inhibit cancer cell migration, decrease tumor development, and response to stress. In contrast, the function of CAFs is impaired in the presence of mutated p53, where they promote cancer cell proliferation. p53 transactivates programmed death-ligand 1 (PD-L1) and its receptor programmed death-1 (PD-1) in cancer cells and normal T cells in response to stress leading to suppression of CD8^+^ T cells

CAFs (cancer-associated fibroblasts) are an essential part of the TME and modulate inflammatory and leukocyte recruitment signals [112]. When CAFs come into contact with cancer cells, they trigger the IFN-β pathway, which interacts with wild-type p53 in fibroblasts to inhibit cancer cell migration and decrease tumor development (Fig. 4) [113, 114]. In contrast to its wild-type counterpart, mutant p53 in cancer cells regulates and inhibits the tumor-suppressive response to IFN-β via inhibiting STAT1 phosphorylation and downstream targets of IFN-β. IFN-β produced by CAFs, in turn, can lower the amounts of mutant p53 RNA in tumors [115] The inflammatory microenvironment can disrupt the equilibrium of this regulatory network, causing a molecular stop that both suppresses and enhances the tumorigenic effects of mutant p53 in cancer cells [116]. Reactivating wild-type p53 activity might be a synergistic opportunity for targeting IFN-related therapy, as the mutational state of p53 is important for targeting IFN-related therapy.

Previous research has shown that the ECM can regulate p53 activity in cultured cells by sending pro-survival signals that reduce p53-evoked apoptotic effects [117]. In recent years, the reciprocal involvement of p53 in ECM regulation has been filled out, particularly in hypoxic situations [118]. The accumulation of reactive oxygen species (ROS) and inflammation are linked to hypoxic tumor environment. In hypoxic settings, hypoxia-inducible factors (HIFs) can enhance the production of pro-angiogenic factors such as vascular endothelial growth factor (VEGF) and platelet-derived growth factor (PDGF) [119]. R273H and R246I p53 mutations cooperate with HIF-1 to regulate transcription of ECM components in non-small cell lung cancer cells, favoring aggressive invasion and poor clinical prognosis [120].

#### *Mutant p53 and* cancer immunology

Despite the fact that p53 mutations are uncommon in immune cells, p53 can impair cell-mediated immunity by creating specific molecular fingerprints in tumor or stromal cells that affect immune cell recruitment and activation [121]. It can also regulate the expression of the class 1 major histocompatibility complex (MHC-1) and the resulting immunological responses [122]. Myeloid-derived suppressor cells are a heterogeneous group of immune cells from the myeloid lineage. MDSCs strongly expand in pathological conditions such as chronic infections and cancer, as a result of an altered hematopoiesis [123]. Lymphocytic penetration, particularly cytotoxic T cells, is hindered when the wild-type p53 pathways are disturbed in ER-negative breast cancer and basal-like breast tumors. Both loss of heterozygosity and p53 mutations are linked to lower incidence of T cell infiltration and a worse prognosis [124]. Multiple genetically altered mouse breast cancer models with p53 loss increased inflammatory Wnt signaling in tumor-associated macrophages, resulting in systemic neutrophilia and finally metastasis [125].

Recent data have indicated that Immunological checkpoints and wild-type p53 are linked. p53 transactivates programmed death-ligand 1 (PD-L1) and its receptor programmed death-1 (PD-1) in cancer cells and normal T cells in response to stress (Fig. 4) [126]. The connection between PD-L1 on tissue and PD-1 on T cells decreases activation signals produced by T cells after antigen recognition, and this immune checkpoint controls inflammation. Tumor amplification of PD-L1, on the other hand, takes advantage of this immune checkpoint mechanism to reduce tumor surveillance and build immunological tolerance [127]. As a result, in some context, mutant p53 might be a useful biomarker for immunotherapy response and might associate with a better prognosis due to distinct immunogenic signals [128].

Wild-type p53 controls Toll-like receptor (*TLR*) gene expression in T-lymphocytes and to a lesser extent in macrophages in a way that is dependent on genetic stress and the host genetic background [129]. Polymorphisms in the p53 response areas of TLR gene promoters, in particular, confer different levels of susceptibility to genetic stress and infection Different levels of vulnerability to genetic stress and infection are conferred by polymorphisms in the p53 response regions of TLR gene promoters, in particular. [130]. The anti-tumor effects of TLR induction become obvious when considering the importance of APC reactivation in the cancer microenvironment, where activated TLR pathways increase immune recognition and action against tumor-antigen carrying cells. On the other hand, TLR expression in tumor cells and surrounding cells is pro-tumorigenic [131]. TLR4 is expressed in several human cancer cell lines, including MDA-MB-231, MCF7, A549, and H1291. In response to LPS treatment, TLR4 activates the p38 MAPK and NF-kB signaling pathways in A549 and H1299 cells. This activation increases cancer immune evasion and resistance to apoptosis by secreting immunosuppressive cytokines such as VEGF, TGF-, and IL-8 [132].

MAPK and NF-kB activation are common threads in TLR-4-expressing colorectal tumors, and it is associated with increasing proliferative capacity, apoptotic resistance, and metastatic potential [133]. In breast cancer, TLR-4 expression has been associated with poor survival and invasiveness [134]. Humans and apes are the only species that have a p53-TLR regulatory axis [135]. This evolutionary gap is significant for considering TLR-mediated cancer treatment since mouse models do not mimic the regulatory axis that is present in humans [136]. p53 mutations not only alter TLR gene expression, but also have a number of additional consequences. TLR3 sensitivity and reactivity to known ligands are affected by these alterations, which modulate type I interferon response and downstream genotoxic-stress-induced apoptosis. This control of TLR3 responsiveness is directly linked to the expression of transcriptionally active or TLR3-enhancing p53 mutants like P151H and R337H, while other mutations might instead inhibit the TLR3-mediated immune response [137].

Macrophages are one of the most prevalent immune cell types in the TME [138]. According to most research, both M1-like and M2-like polarization are often linked to increased levels of p53 expression. High levels of p53 activity function as a brake on M1-like macrophage polarization, avoiding detrimental long-term activation of the inflammatory NF-κB and STAT1 pathways and, as a result, decreased M1-like gene expression over time (Fig. 4) [139]. Exosomal-mediated microRNA transfer is crucial in many cancers, and another mutant-specific GOF of p53 might be relevant as well [140]. Exosomes from R248W and R273H mutant p53-expressing colon cancer cells had a high concentration of miR-1246, a microRNA related to invasiveness and stemness [141]. Mutant p53-expressing tumors can cause comparable non-cell-intrinsic reprogramming of macrophages into TAM-like M2 phenotypes via exosomal microRNA transfer (Fig. 4). These reprogrammed macrophages additionally presented enhanced degradation of the extra cellular matrix and became more invasive when compared with macrophages that were introduced to tumor cells that did not carry any p53 mutation [142].

### P53 and cellular senescence

In the elderly, cellular senescence and the accompanying secretory phenotype (SASP) induce illness. Targeting senescent cells through SASP regulation, or cellular reprogramming could be a new therapeutic path for cancer and age-related illnesses like neurodegeneration, pulmonary fibrosis, and renal failure. The *TP53* gene, encodes 12 or more p53 protein isoforms, regulates cellular senescence. The various p53 isoforms are generated by the use of different transcriptional and translational start sites, as well as alternative mRNA splicing. These shortened p53 isoform proteins play significant roles in cellular senescence, apoptosis, and DNA repair, as well as modulation of full-length p53-mediated cellular senescence, apoptosis, and DNA repair [143].

ELK1 could be targeting the promoters of *TP53* and RB2/P130 [144]. This finding is intriguing because *P53* and *RB2/P130* were identified as the major regulators of senescence in human MSCs [144–146].

In the context of senescence, p53 plays a critical role in deciding the fate of cells, and its activation can be DDR-dependent or DDR-independent [147]. In the first example, replicative stress activates the DNA damage repair cascade by causing telomere erosion, DNA damage, hyperactivation of oncogenes, and inactivation of onco-suppressors (oncogene induced senescence, OIS) [148]. By phosphorylating both p53 and its ubiquitin ligase Mdm2, ATM/ATR activates the p53/p21cip1 axis, resulting in the stability of p53 levels [149].

The role of DDR activation as a necessary and causal element in p53 activation and senescence induction has lately been questioned. Many recent investigations have shown that many OIS routes can really activate p53 without going through the DDR as in fact they might occur throughAKT via downregulation of MnSOD, through the onco-suppressor PTEN depletion, prompting mTORC1 and mTORC2 to bind to p53 instead of MDM2, and MAPK p38 by direct phosphorylation of p53 [149]. These findings, as well as the mechanisms they describe, highlight the critical role of p53 and p53-triggered senescence in the inhibition of carcinogenesis following the occurrence of a first mutation [150].

### Potential of p53 signaling targeting for cancer therapy

Because wild-type p53 is an efficient promoter of apoptosis and senescence [145] in tumor cells, reactivating wild-type characteristics of p53 mutants, which are commonly overexpressed in cancer, is a viable therapeutic strategy.

Several studies have shown that transfection of cancer cells with wild-type p53 expressing plasmids can induce apoptosis and/or growth arrest, implying that a gene therapy method for cancer treatment could be based on restoring normal p53 expression and function. Several clinical research investigations using viral and non-viral vectors delivering p53 genes, alone or combined with other therapeutic agents, have been completed to far [151].

Some tumor-derived mutations that cause wild-type p53 loss-of-function can be restored by other point mutations that help stabilize the p53 protein, indicating that the structural change is reversible [152]. PhiKan083 and PK7088 are small chemicals that bind p53 and generate the Y220C mutant, stabilizing it and boosting the amount of wild-type p53 [153]. Other molecules, including PRIMA-1, PRIMAmet/APR-246, CP-31398, and SCH29074, bind to mutant p53 proteins and interact with DNA binding domains to facilitate proper mutant protein folding and p53 function recovery. The 367–369 Zinc binding domain is required for the proper folding of wild-type p53, whereas zinc binding is absent in the R175H p53 mutant [154, 155].

When zinc is added to the structural mutants G245C and G245D, the wild-type structure is for the most part restored [156] As a consequence, the discovery of the ability of zinc to restore wild-type folding suggest that this technique might be able to restore anticancer drug chemosensitivity in cells harboring mutant p53 proteins [157]. NSC31926, a thiourea metal chelator, is able to restore wild-type p53 function in p53 mutant cell lines, most likely via boosting zinc bioavailability to p53 mutants [158]. Although certain components are designed to selectively inhibit mutant p53, many of them can also interact with and inhibit other members of the p53 family, including p63 and p73. A small compound called RETRA, which was identified in a screening of a drug used to identify stable wt p53, disrupts the interaction between mutant p73 and p53. RETRA increased the release of p53, which inhibited tumor cell survival and xenograft tumor development by activating the p73 gene.

Compounds like APR-246, PK11007, and COTI-2 are promising treatments for patients with trible negative (TN) breast tumours because p53 is mutated in the great majority of them. However,mutant p53 can work as a biomarker in breast cancer, is not clearly defined [159].

Critical Outcome Techonologies Inc.'s (COTI-2) third-generation thiosemicarbazone, as well as particular peptides, have recently been shown to convert reactive mutant p53 protein to a form with wild-type characteristics. These drugs have been demonstrated to have anticancer action in preclinical models expressing mutant p53, which is consistent with the reactivation of mutant p53. Two of these drugs, APR-246 and COTI-2, have made it to clinical trials so far. APR-246 had no major side effects in a phase I/IIa clinical trial. APR-246 is now being tested in patients with advanced serous ovarian cancer in a phase Ib/II trial, while COTI-2 is being tested in patients with advanced gynaecological tumours in a phase I trial. However, whether any mutant p53 reactivating chemical is effective in the therapy of human cancer remains to be seen [160].

Shenzhen SiBiono GeneTech Co. Ltd. produced Gendicine (recombinant human p53 adenovirus), which was approved by the China Food and Drug Administration (CFDA) in 2003 as a first-in-class gene therapy treatment to treat head and neck cancer and went on the market in 2004. Gendicine is a biological therapy that can be administered via intratumoral injection, intracavity infusion, or intravascular infusion. Depending on the cellular stress circumstances, the wild-type (wt) p53 protein expressed by Gendicine-transduced cells is a tumour suppressor that is triggered by cellular stress and promotes cell-cycle arrest and DNA repair, or produces apoptosis, senescence, and/or autophagy. Based on more than 30 published clinical trials and 12 years of commercial use in over 30,000 patients, Gendicine has a proven track record of safety, and when combined with chemotherapy and radiotherapy, it has shown to produce much higher response rates than traditional therapies alone. In addition to head and neck cancer, Gendicine has been used to successfully treat a variety of other cancer kinds and stages. Thirteen published trials with long-term survival data found that Gendicine combination regimens result in considerably longer progression-free survival periods than standard treatments alone. Despite the fact that the p53 gene is mutated in more than half of all human malignancies, the presence of a p53 mutation had no effect on efficacy or long-term survival in Ad-p53-treated patients [161].

## Conclusions

The growing understanding of mutant p53 actions has contributed to the identification of a number of compounds with promising therapeutic potential. However, further experiments are required to fully characterize mutant p53 function in cancer. The fact that mutant p53 might play a role in promoting metastasis – the primary cause of cancer-related mortality – is particularly attractive in terms of possible therapeutic applications. Although many tumors express mutant p53, it is unclear if the many mutations present on this protein have similar activity, and we might have to personalize therapy depending on the presence of a particular mutation rather than only consider whether a cancer express a wild-type vs a mutant p53 mutant.

## Data Availability

All data are included in the manuscript.
